# Endoscopic CO_2_-Laser Surgery for Vocal Cord Cancer

**DOI:** 10.1155/DTE.1.69

**Published:** 1994

**Authors:** J. Czigner, L. Sávay

**Affiliations:** 1 Department of Otorhinolaryngology University Medical School Szeged Hungary; 2 Department of Otolaryngology Albert Szent-Györgyi University Medical School Tisza Lajos krt 111 Szeged H-6725 Hungary

## Abstract

A retrospective study is reported on endoscopic CO_2_-laser microsurgery in 69 patients with histologically verified early vocal cord cancer. A flexible nasopharyngolaryngoscope (STORZ Co) was used for preoperative assessment and occasionally for postoperative follow-up.

Six years of experience with this technique have led to endoscopic cordectomy, previously not accepted as a therapeutic method alone, but which has become the favored method with use of the CO_2_ laser endoscopically. Laser surgery as a therapeutic endoscopic procedure provided successful treatment of early vocal cord cancer in 59 (86%) of the 69 patients. The initial success rate together with “salvage” treatment modalities reached 96% (66/69 patients).

Endoscopic laser surgery resulted in a decrease in voice intensity and phonatory duration from near normal to mildly abnormal. Voice preservation succeeded in 97% of all patients. Thus, the data demonstrate that endoscopic laser surgery is a useful modem method of therapeutic endoscopy for early vocal cord carcinoma.


*Diagnostic and Therapeutic Endoscopy*, Vol. 1, pp. 69–74
Reprints available directly from the publisher
Photocopying permitted by license only

© 1994 Harwood Academic Publishers GmbH
Printed in Malaysia

### Endoscopic $CO_2$-Laser Surgery for Vocal Cord Cancer

J. CZIGNER and L. SÁVAY

*Department of Otorhinolaryngology, University Medical School, Szeged, Hungary*

*(Received September 17, 1993; in final form April 15, 1994)*

A retrospective study is reported on endoscopic $CO_2$-laser microsurgery in 69 patients with histologically verified early vocal cord cancer. A flexible nasopharyngolaryngoscope (STORZ Co) was used for preoperative assessment and occasionally for postoperative follow-up.

Six years of experience with this technique have led to endoscopic cordectomy, previously not accepted as a therapeutic method alone, but which has become the favored method with use of the $CO_2$ laser endoscopically. Laser surgery as a therapeutic endoscopic procedure provided successful treatment of early vocal cord cancer in 59 (86%) of the 69 patients. The initial success rate together with "salvage" treatment modalities reached 96% (66/69 patients).

Endoscopic laser surgery resulted in a decrease in voice intensity and phonatory duration from near normal to mildly abnormal. Voice preservation succeeded in 97% of all patients. Thus, the data demonstrate that endoscopic laser surgery is a useful modern method of therapeutic endoscopy for early vocal cord carcinoma.

**KEY WORDS:** $CO_2$ laser, microsurgical endoscopy, results of cordectomy, vocal cord cancer

### INTRODUCTION

The transoral removal of carcinoma including vocal cord lesions was generally criticized in the US and Europe until the early 1970s (Martin, 1958; Kleinsasser, 1968). Because the fear of tumor dissemination, the procedure was considered unsound by oncologists. There is no doubt that endoscopic treatment of early vocal cord carcinoma is not a new concept; the concept of endoscopic laser excision gained acceptance after 1972 (Strong and Jakó, 1972).

The value and safety of the $CO_2$ laser in laryngeal microsurgery has been well documented for the management of nonmalignant lesions and selected cases of early carcinomas (Jakó, 1972; Davis *et al.*, 1982; McGuirt and Browne, 1991). Sophisticated details of the limitations of laser excision were recognized, and experience in its use resulted in improvement of the indications. Advocates agree that this technique represents a "laser excisional biopsy" for a mid–vocal-cord tumor with no involvement of the anterior commissure, vocal process, ventricle, or subglottic larynx (Shapshay *et al.*, 1990). This limited indication existed to 1990.

Address for correspondence: Jeno Czigner, M.D., Professor and Chairman, Department of Otolaryngology, Albert Szent-Györgyi University Medical School, Tisza Lajos krt 111. H-6725 Szeged.

This report examines the problems associated with the transoral excision of vocal cord carcinoma and laser technology and reports our experience in the excision of this lesion using microendoscopically controlled laser surgery.

### MATERIALS AND METHODS

### Patients

From May 1987 through April 1993, information was obtained on 69 patients admitted to the Otolaryngology Clinic with early glottic carcinoma, Szeged, Hungary, for endoscopic laser surgery. There were 64 men and 5 women. The patients' ages ranged from 37 to 90 years, with an average of 67 years.

### Technology

A large laryngoscope of Kleinsasser or Weerda design (STORZ, Germany) was used for exposure, and a TLS 61 $CO_2$ laser with a visible coaxial helium-neon laser (TUNGSRAM, Hungary) instrument coupled to a Zeiss operating microscope using a 400-mm length with micromanipulator on the laser delivery head was applied. All procedures were performed under general anesthesia. Moist gause or cotton was placed in the subglottic space to prevent inadvertent laser injury to adjacent tissues and the cuff (Figure 3 A,B). The tube was sometimes protected with aluminium foil, although with experience this rarely is necessary now.

The extent of epithelial spread and the depth of tumor invasion were evaluated on the basis of vocal fold motion assessment by clinical examination and preoperative fiberoscopy and vocal fold palpation during operative endoscopy. Laser excisions were made at a power setting of 5 to 15 W in continuous mode as a light scalpel to excise the lesion with appropriate margins as an en bloc resection suitable for histologic examination (Figure 1 A). Therapy for all but two patients consisted of laser excision of the vocal cord lesion, followed by vaporization of adjacent and deeper tissues that appeared doubtful through the operating microscope. Hemostasis was sufficient during the operation in most patients, but there were some cases in which cauterization of the paraglottic artery was necessary.

### Surgical Technique

Familiarization with the practicability of endoscopic laser dissection in relation to the vocal cord lesions lead to classification of the following subgroups of endoscopic cordectomy: 1) Glottic lesions limited to the surface of the true vocal cord, i.e., Tis and exophytic superficial lesions (Fig. 1 A), were dissected submucosally, over the muscle layer, by *superficial* laser cordectomy (Fig. 1 B). The architecture of the vocal cord was preserved because the depth of vaporization was usually superficial in early cases (Fig. 1 C). 2) *Partial* laser cordectomy (Fig. 2 B) was performed for mid–vocal-cord T1a lesions with limited involvement of the soft tissue (Fig. 2 A). 3) *Total* cordectomy was required when patients were found to have invasive carcinoma on one vocal cord into the vocal muscle but without limitation of mobility (Figs. 3 A-B). 4) *Extended* cordectomy was the endoscopic surgical method for any vocal cord cancer with involvement of the anterior commissure as T1b stage, vocal process, or multifocal lesion (Fig. 4).

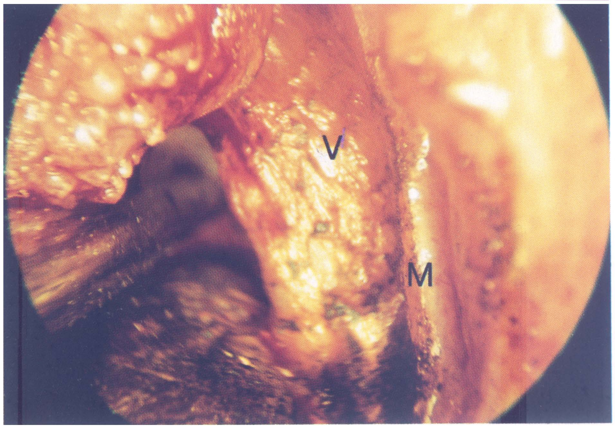

**Figure 1B** Endoscopic intraoperative photograph. Higher magnification of this laser dissection. Note the clear margin after mucosal incision (M) and the preserved vocal muscle layer (V).

### Follow-up

Patients were followed up carefully in the usual fashion for patients with head and neck carcinoma by using office laryngoscopy with either a flexible fiberoptic laryngoscope, a rigid 90° rod lens telescope, or both. One patient had postoperative subcutaneous emphysema, and one patient developed chondritis after extended cordectomy. Some patients had slight edema on the arytenoid fold on the operated side, but none of them required tracheostomy. Postoperative wound care was not necessary. Some patients developed granulation tissue, two requiring post-

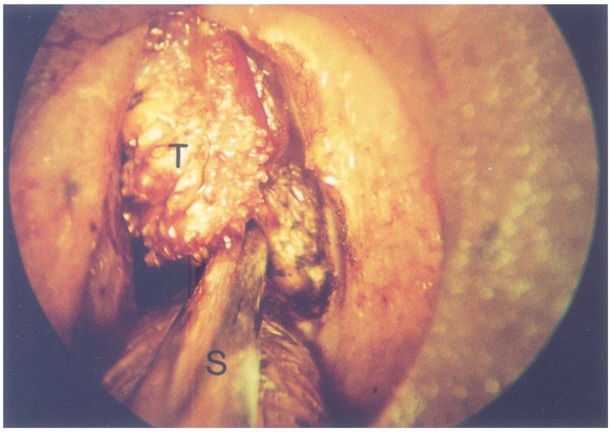

**Figure 1A** Endoscopic intraoperative photograph. A large exophytic superficial cancer on the right vocal cord (T) at the beginning of superficial laser cordectomy (S: suction tube).

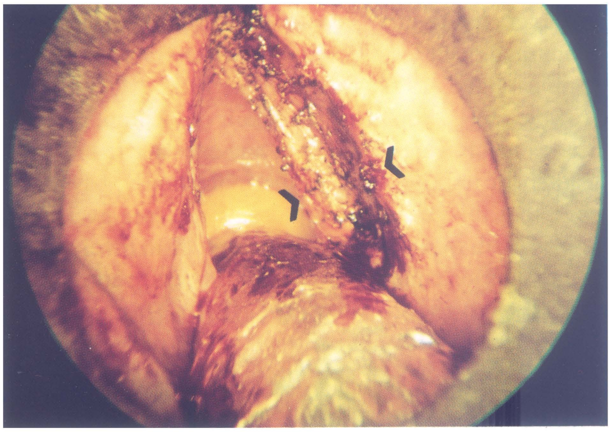

**Figure 1C** Endoscopic intraoperative photograph. Superficial laser cordectomy; the tumor has been resected, completed with slight vaporization on the wound surface (arrows).

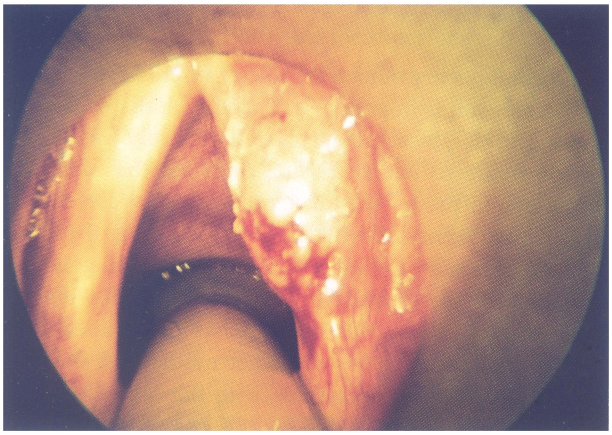

**Figure 2A** Endoscopic photograph. Mid–vocal-cord carcinoma (T1a) on the right.

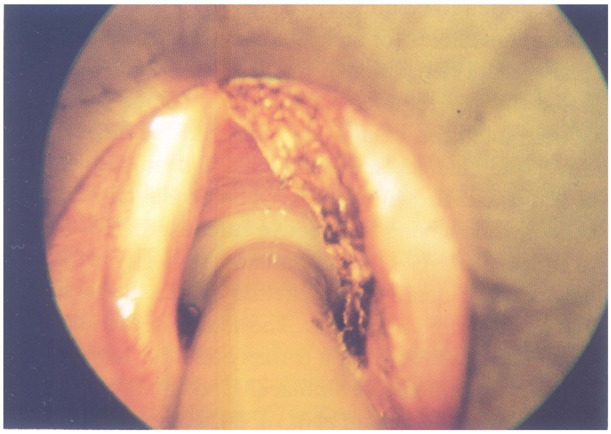

**Figure 2B** Endoscopic photograph. This tumor was ideal for partial laser cordectomy. Resection did not include the deep vocal muscle, vocal process, or anterior commissure.

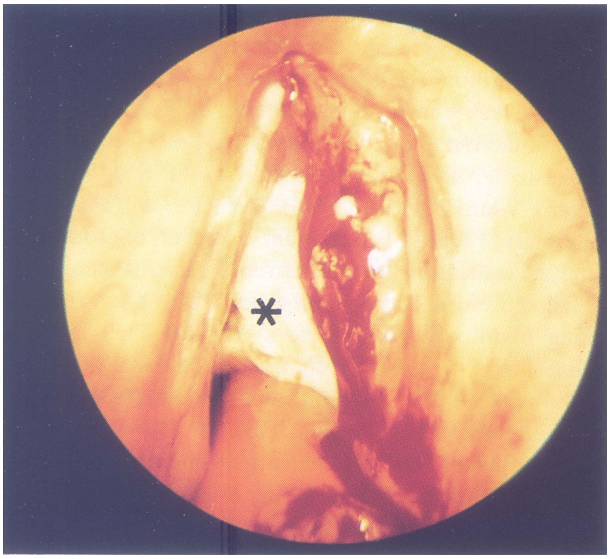

**Figure 3A** Endoscopic photograph. Right vocal cord cancer extended to the anterior commissure (*moist cotton).

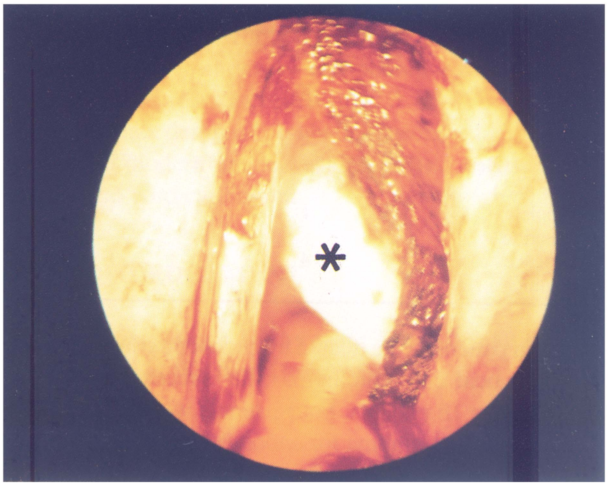

**Figure 3B** Endoscopic photograph. Resection extended to the anterior commissure and vocal process but did not include the subglottic larynx, i.e., total cordectomy (*moist cotton).

operative stripping, one of them four times. Complete healing took 4 to 6 weeks. The follow-up period varied from 6 months to 6 years, with a mean of 3.4 years.

### Results

In this series of 69 patients with untreated vocal cord carcinoma (Table 1), the preliminary oncologic success rate of endoscopic laser excision was 59 (86%) of the 69 patients. All 7 patients with carcinoma in situ and 35 (88%) of the 40 patients with T1a vocal cord carcinoma remained tumor free.

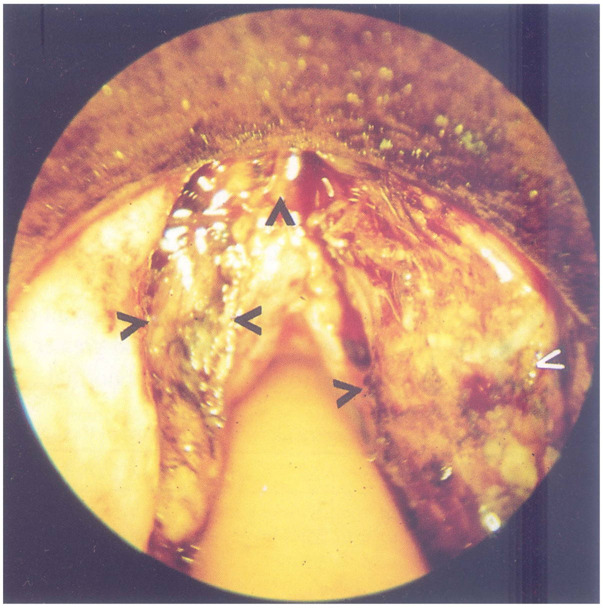

**Figure 4** Extended laser cordectomy after excision of multifocal glottic cancer. Resection extended to the left vocal cord, anterior commissure, and right vocal cord. Note the clear cut margins (arrows).

**Table 1** Endoscopic laser surgery for vocal cord cancer

| | *Tis* | *T1a* | *T1b* | *T2* | *Total* |
|---|---|---|---|---|---|
| Number of patients | 7 | 40 | 18 | 4 | 69 |
| Free of tumor (after single laser exc.) | 7 (100%) | 35 (88%) | 14 (78%) | 3 (75%) | 59 (86%) |
| Incomplete resection | - | 1 | - | - | 1 |
| Recurrence of tumor | - | 4 | 4 | 1 | 9 |

Eighteen patients were treated by endoscopic laser excision for T1b vocal cord carcinomas; the success rate was 14 (78%) of the 18 patients. Four patients were referred with T2 vocal cord carcinoma for endoscopic laser therapy, and 3 of them (75%) remained free of tumor.

Of the 69 patients treated with endoscopic laser resection, most maintained normal-appearing larynges after a single operative procedure. In one patient, the laser resection proved incomplete, and in 8 patients so far a local recurrence has been diagnosed. One other patient has developed a metastasis in the cervical lymph node. One of them had required a single additional laser procedure and did well, with no recurrence. Three of these 10 underwent partial laryngectomies, with no recurrence, but one is a cannula holder because of cicatrization. The remaining 4 patients went on to have radiotherapy. Total laryngectomy was performed for salvage in 2 of these 4 patients, in whom radiation therapy failed. One other patient developed a metastasis in the cervical node after radiotherapy, 31 months following the laser use. Three of the 69 patients in this series died from laryngeal carcinoma.

The initial success rate together with salvage therapy was 66 (96%) of the 69 patients, with voice preservation in 97%. Five patients died later without tumor recurrence, and one patient could not be followed up. The later outcome of the endoscopic treatment along with subsequent management is listed in Table 2.

After endolaryngeal laser surgery, the vocal cord frequently is replaced by an endolaryngeal scar with sufficient phonatory function. We found minimal but consistent differences in voice quality between the lesser and greater resection groups, from normal to rarely mildly abnormal.

**Table 2** Outcome of laser surgery along with subsequent therapy

| *T stage* | *No. of patients* | *NET* | *Died of cancer* | *Died of ICD* |
|---|---|---|---|---|
| Tis | 7 | 6 | - | 1 |
| T1a | 40 | 37 | 2 | 1 |
| T1b* | 17 | 13 | 1 | 3 |
| T2 | 4 | 4 | - | - |
| Total | 68 (100%) | 60 (88%) | 3 (5%) | 5 (7%) |

NET = no evidence of disease; ICD = intercurrent disease

*One patient was lost to follow-up

### DISCUSSION

In all attempts to evaluate the role of lasers in endoscopic laryngology, it is important to maintain realistic expectations. The $CO_2$ laser can be used in a sharply focused excision mode to excise tissue, or it can be used in a vaporization mode, but all neoplasms now are excised rather than vaporized with the laser. Training of the surgeon is necessary to facilitate the understanding and application of lasers effectively. There is certainly nothing magical about the use of the $CO_2$ laser in therapeutic endoscopy, but compared with other modalities, the laser provides a no-touch technique with microprecision and excellent hemostasis.

The preferred method of treatment of laryngeal early vocal cord lesion is controversial. Radiation therapy has been reported to be quite effective (79%–93%) and is the treatment of choice for many authors (Hendrickson, 1985; Viani *et al.*, 1991; Van den Bogaert, 1993). However, radiation therapy requires a rather long course of therapy with the inclusion of normal tissue and secondary long-term tissue changes such as edema and mucosal drying (McGuirt and Browne, 1991). Open partial laryngeal resection, while having an effective cure rate of 70% to 94% (Rothfield *et al.*, 1989), causes major morbidity in the form of poor vocal quality and function due to the inordinate amount of normal tissue loss. The abundant literature may not be reviewed here, but it is likely that the survival rates for the two methods are similar (Mendenhall *et al.*, 1988; Glanz *et al.*, 1989; Viani *et al.*, 1991; Czigner, 1993).

The advantages of laser excision as opposed to radiotherapy as a treatment for T1 vocal cord carcinoma are well known. This treatment option allows one-session therapy with an endoscopic approach, less morbidity, and fewer side effects, and cost-effectiveness as compared with the prolonged hospitalization for open surgery or a 5- to 6-week course of radiotherapy. Excellent success rates in the range of 89% to 96% have been reported by Blakeslee *et al.* (1984), Ossoff *et al.* (1985), Koufman (1986), Wetmore *et al.* (1986), Steiner *et al.* (1991), and Thumfart (1993). It is important, however, not to overextend the indications of this extremely useful technology to larger tumors. These tumors should never be vaporized using histologic control from random deep biopsies (Shapshay *et al.*, 1990). It should be noted that the line of section by the $CO_2$ laser does not prevent assessment of the margins of resection (Fig. 1 B).

Prerequisites for this laser surgery include the following. 1) Patients must be medically eligible for endoscopic surgery using suspension laryngoscopy; 2) Adequate exposure must be obtained so that the entire lesion can be visualized; and 3) The lesion must be confined to the true vocal fold. Studies of the anatomic limitations of laser cordectomy (Davis, 1990) and the superior exposure after vestibulectomy (Kashima *et al.*, 1993) allowed improvement of the assessment of the paraglottic space for laser surgery and accuracy in follow-up examinations.

It seems reasonable to define different types of endolaryngeal resections (Kaufman, 1986; Eckel and Thumfart, 1992). Our 6 years of experience with this technique also led us to form different subgroups of endoscopic cordectomy. This procedure, not earlier accepted as a therapeutic method alone, has now become the favored method with use of the $CO_2$ laser endoscopically. Excellent control of early vocal cord carcinoma is possible with one endoscopic treatment. In our series, Tis and T1a vocal cord cancers were successfully cured using this method alone in 89% (42/47) and for all early vocal cord cancers in 59 (86%) of the 69 patients.

Laser surgery can be extended (Steiner *et al.*, 1991; Eckel and Thumfart, 1992) to extensive superficially growing carcinomas, and so it seems to be an effective approach to the curative treatment of T1b and T2 tumors of the glottis with a fairly high recurrence rate (Hirano and Hirade, 1988; Inouye and Tanabe, 1988). This was illustrated in our patients, in whom stages T1b and T2 success rates were 78% and 75%, respectively. Deeply infiltrating carcinomas especially at the anterior commissure might be less suitable for laser surgery (Davis *et al.*, 1982; Shapshay *et al.*, 1990).

The rate of recurrence observed to date is not higher than would be expected after conventional partial larynx resection or after radiotherapy. One of the most important advantages of endoscopic laser surgery for this lesion may be that all other treatment options are still available should the tumor recur in a more aggressive form or should a second primary lesion develop. This is underlined by our results that the initial success rate together with "salvage" treatment modalities achieved 96%. Laser cordectomy failures can be saved by salvage surgery or postoperative radiotherapy, but we agree with Steiner (1991) and Eckel and Thumfart (1992) that combined laser excision and radiotherapy of T1 glottic carcinoma must be regarded as overtreatment for these tumors. We agree with Shaphsay (1990) that radiotherapy should be saved for those patients who are not good risks for general anesthesia and for patients larger, more invasive T1 vocal cord carcinoma, specifically those involving the anterior commissure. If deep extension into the cartilage is noted, open partial laryngectomy of frontolateral type, etc., may be chosen for definitive treatment. However, if the vocal cord mobility is limited as seen with larger T1 or T2 vocal cord carcinomas better success can be obtained with open partial laryngectomy than with radiotherapy.

Finally, as documented by Koufman (1986), the vocal quality and function after a short healing period are excellent in these patients, because excess normal tissue is not sacrificed. Endoscopic laser cordectomy resulted in a decrease in voice intensity and phonatory duration, but voice preservation succeeded in 97% of all our patients. Thus, our data demonstrate that endoscopic laser surgery is a useful modern method of therapeutic endoscopy for early vocal cord carcinoma.

### ACKNOWLEDGEMENT

This study was supported by grant T-103/1990/ETT from the Ministry of Welfare, Hungary.

### REFERENCES

Blakeslee D., Vaughan C. W., Shapshay S. M. *et al.* (1984) Excisional biopsy in the selective management of T1 glottic cancer: a three-year follow-up study. *Laryngoscope*, 94:488–494.

Czigner J. (1993) Analysis of treatment failures of early glottic carcinoma. In: Johnson J. T., Didolkar M. S. (eds.): *Head and Neck Cancer, Volume III*. Excerpta Medica, Elsevier Science Publishers, Amsterdam, 197–204.

Davis, R. K., Jako G. J., Hyams V. J., *et al.* (1982) The anatomical limitations of $CO_2$ laser chordectomy. *Laryngoscope* 92:980–984.

Davis R. K. (1990) *Lasers in Otolaryngology, Head and Neck Surgery*. W.B. Saunders Co., Philadelphia.

Eckel H. E., Thumfart W. F. (1992) Laser surgery for the treatment of larynx carcinomas: indications, techniques, and preliminary results. *Ann Otol Rhinol Laryngol* 101:113–118.

Glanz H., Kimmich T., Eichhorn T., *et al.* (1989) Behandlungsergebnisse bei 584 Kehlkopfkarzinomen an der Hals- Nasen-Ohrenklinik der Universität Marburg *HNO* 37:1–10.

Hendrickson F. R. (1985) Radiation therapy of larynx cancers. *Cancer* 55:2058–2061.

Hirano M., Hirade Y. (1988) $CO_2$ laser for treating glottic carcinoma. *Acta Otolaryngol* [Suppl] (Stockh), (suppl 458):154–157.

Inouye T., Tanabe T. (1988) $CO_2$ laser management of laryngeal carcinoma. *Acta Otolaryngol* [Suppl](Stockh), (suppl 458):158–162.

Jako G. J. (1972) Laser surgery of vocal cords. *Laryngoscope* 82:2204–2209.

Kashima H. K., Lee D. J., Zinreich S. J. (1993) Vestibulectomy in early vocal fold carcinoma. In: Johnson J. T., Didolkar M. S. (eds.): *Head and Neck Cancer, Volume III*. Excerpta Medica, Elsevier Science Publishers, Amsterdam, 181–189.

Kleinsasser O. (1968) *Mikrolaryngoskopie und endolaryngeale Mikrochirurgie*. Schattauer, Stuttgart.

Koufman, J. A. (1986) The endoscopic management of early squamous carcinoma of the vocal cord with the carbon dioxide surgical laser: clinical experience and a proposed subclassification. *Otolaryngol Head Neck Surg* 95:531–537.

McGuirt W. F., Browne J. D. (1991) Management decision in laryngeal carcinoma in situ. *Laryngoscope* 101:125–129.

Martin H. (1958) *Surgery of the Head and Neck Tumors*. Harper & Row, New York.

Mendenhall W. M., Parsons J. T., Stringer S. P., *et al.* (1988) T1–T2 vocal cord carcinoma: a basis for comparing the results of radiotherapy and surgery. *Head Neck Surg* 10:373–377.

Ossoff R. H., Sisson G. A., Shapshay S. M. (1985) Endoscopic management of selected early vocal cord carcinoma. *Ann Otol Rhinol Laryngol*, 94:560–564.

Rothfield R. E., Johnson J. T., Myers E. N. *et al.* (1989) The role of hemilaryngectomy in the treatment of T1 vocal cord cancer. *Arch Otolaryngol Head Neck Surg*; 116:677–680.

Shapshay S. M., Hybels R. L., Bohigian R. K. (1990) Laser excision of early vocal cord carcinoma: indications, limitations and precautions. *Ann Otol Rhinol Laryngol* 99:46–49.

Steiner W., Aurbach G., Ambroch P. (1991) Minimally invasive therapy in otorhinolaryngology and head and neck surgery. *Min Inv Ther* 1:57–70.

Steiner W., Iro H., Petsch S. *et al.* (1991) Lasermikrochirurgische Behandlung von Larynxkarzinome (pT2-4) Darstellung der Langzeitergebnisse. In: Dühmke E., Steiner W., Reck R. (eds.): *Funktionserhaltende Therapie des fortgeschrittenen Larynxkarzinoms*. Thieme Verlag, Stuttgart, 80–91.

Strong M. S., Jako G. J. (1972) Laser surgery in the larynx: early clinical experience with continuous $CO_2$ laser. *Ann Otol Rhinol Laryngol* 81:791–798.

Thumfart W. F. (1993) Early cancer of the larynx. Laser-surgery of larynx-carcinomas: indications, techniques and follow-up. In: Johnson J. T., Didolkar M. S. (eds.): *Head and Neck Cancer, Volume III*. Excerpta Medica, Elsevier Science Publishers, Amsterdam, 215–222.

Van den Bogaert W. (1993) What is the treatment of choice in early glottic cancer? In: Johnson J. T., Didolkar M. S. (eds.): *Head and Neck Cancer, Volume III*. Excerpta Medica, Elsevier Science Publishers, Amsterdam, 223–227.

Viani L., Stell P. M., Dalby J. E. (1991) Recurrence After Radiotherapy for Glottic Cancinoma. *Cancer* 67:577–584.

Wetmore S. J., Key N. J., Suen J. Y. (1986) Laser therapy for T1 glottic carcinoma of the larynx. *Arch Otolaryngol Head Neck Surg*, 112:853–855.

